# Reactivation of Hepatitis B After Ibalizumab Therapy for Multidrug-Resistant Human Immunodeficiency Virus

**DOI:** 10.14309/crj.0000000000000594

**Published:** 2021-05-19

**Authors:** Alyson Kaplan, Elliott DeHaan, Charles Maltz

**Affiliations:** 1 Division of Gastroenterology and Hepatology, Department of Medicine, Weill Cornell School of Medicine, New York Presbyterian, New York, NY; 2 Division of Infectious Disease, Department of Medicine, Weill Cornell School of Medicine, New York Presbyterian, New York, NY

## Abstract

Despite the decreasing morbidity associated with the human immunodeficiency virus (HIV), a large percentage of persons with HIV have at least 1 drug resistance mutation. Ibalizumab, a recently approved drug, targets multidrug-resistant HIV. We present a case of reactivation of hepatitis B after initiation of ibalizumab therapy.

## INTRODUCTION

With the advent of antiretroviral therapy (ART), both the morbidity and mortality associated with the human immunodeficiency virus (HIV) have declined. However, there remains a large percentage of persons with HIV who have at least 1 drug resistance mutation.^1^ Because the virus is often not suppressed in this population, it is necessary to develop new drugs to target different viral mechanisms. One of these drugs is ibalizumab, a recombinant humanized IgG4 monoclonal antibody to CD4 which was approved by the Food and Drug Administration in 2018.^2^ Although the drug has generally been deemed safe, we present the case of reactivation of hepatitis B after its initiation.

## CASE REPORT

**Figure 1.**
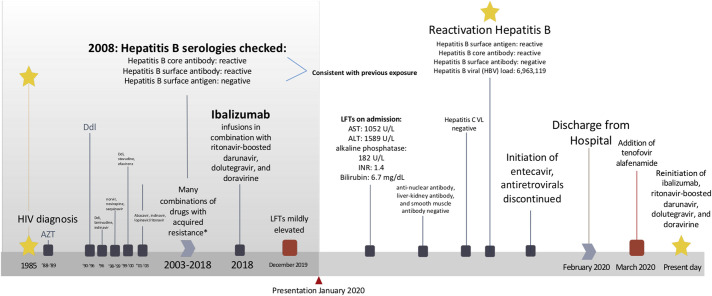
Timeline from the patient's diagnosis with human immunodeficiency virus to this day. *Resistance mutations: NRTIs (M41L, D67G, L74V, M184V, L210W, T215Y, and K219N), NNRTIs (A98G, K103N, V108I, E138A, V179T, Y181C, and F227L), PIs (L10V, L24I, L33F, K43T, I47V, G48V, I54L, Q58E, A71I, V82A, I84V, and L90M), integrase strand transfer inhibitors (Q148H G140S), acquired resistance to entry inhibitors (enfuvirtide), Trofile phenotype = D/M virus. ALT, alanine aminotransferase; AST, aspartate aminotransferase; AZT, zidovudine; DdI, didanosine; INR, international normalized ratio; LFTs, liver function tests.

A 53-year-old man with multidrug-resistant HIV (diagnosed in 1985), latent tuberculosis, and previous exposure to hepatitis B presented from the clinic with jaundice and elevated liver enzymes. The patient had been on several ART regimens. After years of suboptimal adherence, he acquired resistance to all nucleoside reverse transcriptase inhibitors, most non-nucleoside reverse transcriptase inhibitors, all protease inhibitors, several integrase strand transfer inhibitors, entry inhibitors, and to dual/mixed tropic virus (Figure 1).

One year before presentation, he had been initiated on a salvage regimen of ibalizumab infusions in combination with ritonavir-boosted darunavir, dolutegravir, and doravirine, at which time his CD4 count was 88 cells/μL, with an HIV-1 ribonucleic acid 163,000 copies/mL. Within approximately 6 weeks, he achieved a CD4 295 cells/μL, with an HIV-1 ribonucleic acid 191 copies/mL, reaching <20 copies/mL by month 6. His CD4 peaked at 324 cells/μL after 9 months. Concerning his hepatitis B history, serologies checked in 2008 were consistent with previous hepatitis B virus (HBV) immunity (surface antigen [sAg] negative, surface antibody positive, and core antibody positive). HBV serologies were not repeated since that time, given normal liver function tests and no new exposures. Liver tests drawn 6 weeks before presentation were notable for slightly elevated aspartate aminotransferase (AST) of 60 and alanine aminotransferase (ALT) of 97, but were otherwise normal.

On presentation, scleral icterus and jaundice were noted on physical examination. Laboratory workup revealed AST 1,052 U/L, ALT 1,589 U/L, alkaline phosphatase 182 U/L, INR 1.4, bilirubin of 6.7 mg/dL, a CD4 188 cells/μL, and an HIV viral load of 76 copies/mL. An abdominal ultrasound demonstrated an unremarkable sonographic appearance of the liver. The patient reported no history of significant alcohol use, new or over-the-counter medications, or sexual exposures. Autoimmune markers, hepatitis C viral load, and hepatitis D antibody were negative. HBV serologies were drawn and were notable for sAg and core antibody (IgM) positivity with surface antibody negativity. An HBV viral load resulted at 6,963,119 IU/mL, consistent with reactivation of hepatitis B. He was started on entecavir, and all ARTs were stopped. His liver enzymes peaked with an AST 2,014 U/L, an ALT 1,557 U/L, and a bilirubin 6.9 mg/dL.

One month after presentation, the patient was switched from entecavir to combination emtricitabine/tenofovir alafenamide to better treat both his HBV and HIV. Ibalizumab along with his other ARTs were recently readded as this was the only combination that had been successful at suppressing his HIV. Presently, his HBV viral load is 181 IU/mL, and his liver function has normalized.

## DISCUSSION

We describe the case of reactivation of HBV after initiation of ibalizumab, a drug with a unique mechanism of action for use in patients with multidrug-resistant HIV. HIV typically enters the host's cell membrane in a multistep process. The virus contains glycoprotein 120 (gp120) which binds to CD4+ T-cell receptors on the host cell and leads to a conformational change that then allows gp120 to interact with host CCR5/CXCR4 coreceptors. This interaction then leads to HIV fusion entry.^3^ Ibalizumab is believed to inhibit the conformational changes in the CD4/gp120 complex that allows binding to CXCR4, thus inhibiting HIV fusion entry.^4^ Notably, the CD4 T-cell receptor that is the target of ibalizumab is believed to be distant from the binding site of major histocompatibility complex class II molecules, thereby supposedly preventing an major histocompatibility complex II–mediated immune response after binding. This would imply that it should not be immunosuppressive.^5,6^ In addition, ibalizumab is not believed to deplete CD4+ T cells secondary to its low affinity for C1q and for a cellular cytotoxic-dependent response.^7^ These properties make it a very desirable drug.

In our patient, however, we believe that the introduction of ibalizumab led to the reactivation of HBV, despite the fact that it should not suppress immunity. This could have happened by several mechanisms. One is by immune reconstitution inflammatory syndrome, which has been previously described with ART, including ibalizumab, when a patient presented with progressive multifocal leukoencephalopathy.^7^ Immune reconstitution inflammatory syndrome normally occurs around the time of initiation of a drug, when a patient's CD4+ T-cell count rises quickly, but it can also occur up to 2 years after an offending medication is discontinued.^8^ Our patient's immune system had already reconstituted by the time of hepatitis flare, but the possibility of immune reconstitution inflammatory syndrome is still present. Second, patients with resolved HBV infection could have occult infection with persistent viremia despite the absence of circulating HBsAg.^9^ Third, although very rare, HBV superinfection with different HBV genotypes can cause acute exacerbations in hepatitis B carriers.^10^

Finally, although seroconversion to positive HBsAg has not been previously described with ibalizumab, there are other monoclonal antibody class of drugs, including rituximab, obinutuzumab, ocrelizumab, and brentuximab where there is a well-documented risk of reactivation of hepatitis B.^11^ For these reasons, patients who are HBsAg-negative and anti-HBc-positive should be monitored for HBV reactivation during and after completion of treatment with ibalizumab. Reactivation of hepatitis B or any serious hepatic adverse event in the setting of ibalizumab has not been previously reported. In drug trials with ibalizumab, liver enzyme elevations occurred in up to 25% of patients, but these were usually mild or self-limited.^12^ Caution should be exercised in prescribing ibalizumab in a patient with previous hepatitis exposure without appropriate prophylaxis.

## DISCLOSURES

Author contributions: A. Kaplan wrote the manuscript and revised the manuscript for intellectual content. E. DeHaan and C. Maltz revised the manuscript for intellectual content. C. Maltz is the article guarantor.

Financial disclosure: None to report.

Informed consent was obtained for this case report.

## References

[1] Wensing AM Calvez V Ceccherini-Silberstein F. Update of the drug resistance mutations in HIV-1. Top Antivir Med. 2019;27(3):111–21. 31634862 PMC6892618

[2] Iacob SA Iacob DG. Ibalizumab targeting CD4 receptors, an emerging molecule in HIV therapy. Front Microbiol. 2017;8:2323. 29230203 10.3389/fmicb.2017.02323 PMC5711820

[3] Kuritzkes DR. HIV-1 entry inhibitors: An overview. Curr Opin HIV AIDS. 2009;4:82–7. 19339945 10.1097/COH.0b013e328322402e PMC2753507

[4] Toma J Weinheimer SP Stawiski E. Loss of asparagine-linked glycosylation sites in variable region 5 of human immunodeficiency virus type 1 envelope is associated with resistance to CD4 antibody ibalizumab. J Virol. 2011;85:3872–80. 21289125 10.1128/JVI.02237-10 PMC3126132

[5] Moore JP Sattentau QJ Klasse PJ Burkly LC. A monoclonal antibody to CD4 domain 2 blocks soluble CD4-induced conformational changes in the envelope glycoproteins of human immunodeficiency virus type 1 (HIV-1) and HIV-1 infection of CD4+ cells. J Virol. 1992;66:4784–93. 1378510 10.1128/jvi.66.8.4784-4793.1992 PMC241306

[6] Reimann KA Burkly LC Burrus B Walte BCD Lord CI Letvin NL. In vivo administration to rhesus monkeys of a CD4-specific monoclonal antibody capable of blocking AIDS virus replication. AIDS Res Hum Retroviruses. 1993;9:199–207. 8471310 10.1089/aid.1993.9.199

[7] Beccari MV Mogle BT Sidman EF Mastro KA Asiago-Reddy E Kufel WD. Ibalizumab, a novel monoclonal antibody for the management of multidrug-resistant HIV-1 infection. Antimicrob Agents Chemother. 2019;63(6):e00110–9. 30885900 10.1128/AAC.00110-19 PMC6535568

[8] Manegold C Hannoun C Wywiol A. Reactivation of hepatitis B virus replication accompanied by acute hepatitis in patients receiving highly active antiretroviral therapy. Clin Infect Dis. 2001;32(1):144–8. 11118394 10.1086/317535

[9] Di Bisceglie AM Lok AS Martin P Terrault N Perrillo RP Hoofnagle JH. Recent US food and drug administration warnings on hepatitis b reactivation with immune-suppressing and anticancer drugs: Just the tip of the iceberg? Hepatology. 2015;61(2):703–11. 25412906 10.1002/hep.27609 PMC5497492

[10] Kao JH Chen PJ Lai MY Chen DS. Acute exacerbations of chronic hepatitis B are rarely associated with superinfection of hepatitis B virus. Hepatology. 2001;34:817–23. 11584381 10.1053/jhep.2001.28188

[11] Law MF Ho R Cheung CK. Prevention and management of hepatitis B virus reactivation in patients with hematological malignancies treated with anticancer therapy. World J Gastroenterol. 2016;22(28):6484–500. 27605883 10.3748/wjg.v22.i28.6484 PMC4968128

[12] Ibalizumab. Injury, LiverTox: Clinical and Research Information on Drug-Induced Liver. Bethesda, MD: National Institute of Diabetes and Digestive and Kidney Diseases. 31643176

